# Neuroprotective Effects and Mechanisms of Tea Bioactive Components in Neurodegenerative Diseases

**DOI:** 10.3390/molecules23030512

**Published:** 2018-02-25

**Authors:** Shu-Qing Chen, Ze-Shi Wang, Yi-Xiao Ma, Wei Zhang, Jian-Liang Lu, Yue-Rong Liang, Xin-Qiang Zheng

**Affiliations:** Tea Research Institute, Zhejiang University, 866 Yuhangtang Road, Hangzhou 310058, China; 21516096@zju.edu.cn (S.-Q.C.); fdlete@126.com (Z.-S.W.); 21516098@zju.edu.cn (Y.-X.M.); 21516111@zju.edu.cn (W.Z.); jllu@zju.edu.cn (J.-L.L.)

**Keywords:** neuronal degeneration, Parkinson’s disease, Alzheimer’s disease, polyphenols, theanine, caffeine, theaflavins

## Abstract

As the population ages, neurodegenerative diseases such as Parkinson’s disease (PD) and Alzheimer’s disease (AD) impose a heavy burden on society and families. The pathogeneses of PD and AD are complex. There are no radical cures for the diseases, and existing therapeutic agents for PD and AD have diverse side effects. Tea contains many bioactive components such as polyphenols, theanine, caffeine, and theaflavins. Some investigations of epidemiology have demonstrated that drinking tea can decrease the risk of PD and AD. Tea polyphenols can lower the morbidity of PD and AD by reducing oxidative stress and regulating signaling pathways and metal chelation. Theanine can inhibit the glutamate receptors and regulate the extracellular concentration of glutamine, presenting neuroprotective effects. Additionally, the neuroprotective mechanisms of caffeine and theaflavins may contribute to the ability to antagonize the adenosine receptor A_2A_R and the antioxidant properties, respectively. Thus, tea bioactive components might be useful for neuronal degeneration treatment in the future. In the present paper, the neuro protection and the mechanisms of tea and its bioactive components are reviewed. Moreover, the potential challenges and future work are also discussed.

## 1. Introduction

With the accelerated aging population in the world, the prevalence of neurodegenerative diseases such as Parkinson’s disease (PD) and Alzheimer’s disease (AD) is increasing over time. The World Alzheimer Report has shown that the number of people with dementia worldwide was 46.8 million in 2015 and it will be 131.5 million in 2050 [1]. Over 80% of dementias in the world are caused by AD, which imposes a heavy burden on society and family [2]. The occurrence of AD is affected by many factors, including genetic components, oxidative stress, inflammation, neurofibrillary tangle accumulation, amyloid beta peptide (Aβ), mitochondrial dysfunction, hormone imbalances, mitotic disorders, and so on [3]. The pathogenic mechanisms of AD have not been fully clarified, and current treatment is mainly through drugs acting on different neurotransmitter systems. Existing drugs are mainly receptor antagonists of N-methyl D-aspartate (NMDA) and inhibitors of acetylcholinesterase, which have low efficacy and side effects. PD is the second most common neurodegenerative disease in elderly people. It is characterized by a large number of non-motor and motor symptoms, and the reason may be that the synthesis of dopamine is reduced after pathological changes in the substantia nigra [4,5]. The pathogenic mechanisms of PD involve genetic mutations, oxidative stress, mitochondrial respiration defects, inflammation, abnormal protein aggregation, and environmental factors [6,7,8]. Treatment for PD mainly aims at alleviating its clinical symptoms. Some therapeutic agents including rotigotine, levodopa, trastal, selegiline, and ropinirole have been used to treat PD patients due to their relatively high efficacy, although some adverse effects remain [9].

Tea is a popular beverage consumed daily in more than 160 countries. It is well-known for possessing polyphenols, theanine, caffeine, and other natural bioactive components, which are very important for the taste and flavor of tea. For instance, polyphenols, caffeine, and theanine give astringent, bitter, and fresh tastes to tea cream, respectively. In addition, aroma is an important factor affecting tea sensory quality, and more than 600 volatiles have been identified from the aroma of tea [10]. Additionally, tea volatiles generated from Maillard reactions could decrease brainwave distribution, relieve stress, and have sedative effects, but the mechanisms have not been made clear yet [11,12,13]. During the last decade, many studies have proven that tea has neuroprotective functions. Furthermore, compared with western medicine, the neuroprotective effects of tea have the advantages of multi-target, non-toxic, and good synergistic effects. The effects of the polyphenols, theanine, caffeine, and theaflavins in the tea production to neuro function have attracted much attention. In the present paper, the aim is to give a narrative review providing comprehensive understanding of the neuroprotective effects and mechanisms of tea bioactive components, including tea polyphenols, theanine, caffeine, and theaflavins. Furthermore, challenges and future expectations are also discussed in this review.

## 2. Epidemiological Evidence

Many population-based cohort investigations have demonstrated that drinking tea is related to a lower risk of cognitive impairment. A follow-up study for 5.7 years involving 13,645 Japanese over 65 years old showed that green tea consumption significantly reduced the risk of dementia [14]. A study among 278 PD patients revealed that the onset of PD was delayed by 7.7 years when tea consumption was more than 3 cups/day [15].

A series of population-based cross-sectional studies have indicated that drinking tea was related to reduced impairment to cognition. Studies showed that regular consumption of tea, particularly black tea, oolong tea, or green tea was conducive to attention, balance, gait, and basic activities of daily living [16,17]. However, another study of 1003 Japanese aged ≥70 years showed that these people needed higher consumption of green tea [18].

Many meta-analysis studies have shown that tea intake might have neuroprotective effects. Data from 26 previous observations including 52,503 participants showed that daily tea consumption could significantly lower the risk of cognitive decline, cognitive impairment, and mild cognitive impairment in elderly [19]. A study including 34,4895 participants showed that consumption increases of caffeine (200 mg/day) and tea (2 cups/day) decreased PD risk by 17% and 26%, respectively [20].

Although there are many epidemiological studies showing that consumption tea is related with the neuroprotective effects, several inconsistent results are also reported. For instance, a survey including 1409 participants (71% ages 65–79 years) showed that drinking coffee was related to the prevention of AD, while drinking tea had no such function [21]. That might partially be explained by the relatively uncommon tea drinking among the participants, of which only 61 had dementia (48 with AD), which might significantly influence the results. In addition, it is not negligible that people in the survey area may prefer to drink coffee rather than drink tea. Furthermore, there is no relation between tea consumption and AD, and the heterogeneity may be caused by a variety of many factors such as age, gender, and lifestyles [19]. For example, increasing evidence indicates that diabetes mellitus may increase the risk of AD [22]. Finally, it is worth mentioning that most of the epidemiological data are from Asia, however, some evidence obtained from USA, Sweden, and Finland [20] also showed the applicability of tea on neuroprotection to other populations. In summary, tea may have neuroprotective effects, and Table 1 summarizes the epidemiological studies in the last decade.

## 3. Neuroprotective Effects and Mechanisms of Tea Polyphenols

Tea polyphenols (TPs) are complexes of many phenolic compounds, accounting for 24–36% (dry weight) of tea fresh leaves. After processing, tea is divided into unfermented tea (green tea, white tea), semi-fermented tea (oolong tea), fully fermented tea (black tea), and post-fermented tea (dark tea) based on the degree of fermentation. Unfermented teas contain more catechins, while fully fermented tea and post-fermented tea contain more theaflavins and thearubigins. TPs are one of the main bioactive substances in tea. Catechins and their derivatives are the predominant TPs in unfermented teas, comprising 60–80% of total TPs, which include epigallocatechin gallate (EGCG), epigallocatechin (EGC), epicatechin (EC), and epicatechin-3-gallate (ECG). Among them, the most active and abundant compound is EGCG. Studies have shown that TPs, particularly EGCG, have antioxidant, anti-inflammatory, anti-microbial, anti-cancer, and neuroprotective properties [23,24]. In the last decade, the neuroprotective effects and mechanisms of TPs have attracted much more attention. Prior research has revealed that EGCG can cross the blood brain barrier, and its neuroprotective effect has been demonstrated in both in vivo and in vitro studies [25,26,27].

### 3.1. Anti-Oxidative Stress

The brain, as the most oxygen-consuming organ in the human body, is easily damaged by oxidative stress, such as various free radicals and redox-active metals [28]. TPs are natural antioxidants extracted from tea with radical eliminating capacity. In addition, EGCG has been reported to scavenge free radicals by directly or indirectly blocking free radical chain reactions, activating intracellular antioxidant enzyme activity [29], chelating metal ions [30], reducing lipid peroxidation [31], and releasing intracellular calcium [32]. Additionally, EGCG protects neuronal cells against oxidative stress-induced death and apoptosis [30].

A series of studies in both in vivo and in vitro on PD or AD have demonstrated that TPs have the neuroprotective effects through their anti-oxidative properties. Studies have shown that catechins can significantly attenuate 6-hydroxydopamine (6-OHDA)-induced oxidative stress in rats and SH-SY5Y cells, and the mechanisms may be associated with increasing glutathione and tyrosine hydroxylase levels, reducing reactive oxygen species (ROS) contents, as well as regulating ROS-(nitric oxide) NO pathways [33,34,35]. Chronic administration of catechins (0.05%) (the equivalent human dose is 8.79 mg/kg) could prevent oxidative stress-induced brain aging, which was related to an increase in the activities of glutathione peroxidase and superoxide dismutase in the serum of aged mice [36]. 1-methyl-4-phenyl-1,2,3,6-tetrahydropyridine (MPTP) is a neurotoxin that can cause symptoms similar to PD by destroying neurons which produce dopamine in the substantia nigra. EGCG can reduce MPTP-induced oxidative stress damage to PD mice, and its mechanism may be associated with inhibiting the expression level of inducible nitric oxide synthase (iNOS) [37]. As well, EGCG can alleviate hypoxic-ischemic-induced brain injury by reducing iNOS activity [38], significantly attenuating the production of Aβ, and reducing oxidative stress by suppressing the expressions of β-site amyloid precursor protein-cleaving enzyme 1 mRNA and protein [39].

### 3.2. Modulation of Cell Signaling Pathways

A large amount of evidence has indicated that TPs exert a neuroprotective role in neurodegenerative diseases through antioxidation and the regulation of signaling pathways. Indeed, it has been demonstrated that a variety of intracellular signaling pathways play an important role in the neuroprotective effect of EGCG, including phosphatidylinositide 3 kinase (PI3K)/AKT signaling pathways, mitogen activated protein kinases (MAPKs), and protein kinase C (PKC). PKC is serine-threonine kinase, composed of three types of isoforms: conventional (α, βI, βII, γ), novel (δ, ε, θ, η, μ), and atypical (λ, ζ), is critical for regulating cell survival and death, cell metabolism, and differentiation and transcription [40,41,42,43]. In addition, PKC is related to the amalgamation of different kinds of memories [44,45]. In support of this, activated PKC has been shown to prevent Aβ-induced toxic effects on hippocampal neurons [46]. Thus, the activation of PKC may serve as a treatment of the aging brain, such as in AD [47,48]. There are multiple studies showing that TPs might play a neuroprotective role by attenuating PKC signaling pathways. Some studies have reported that the neuroprotection mechanisms of EGCG against cell deaths induced by Aβ and 6-OHDA involve the activation of PKC in PC12 and SH-SY5Y cells [49,50]. Moreover, a previous study showed that EGCG may promote the degradation of Bad protein associated with apoptosis by activating the PKC pathway, thereby increasing cell survival rate and providing neuroprotection [51]. Accordingly, EGCG (maximal effect 1 μM) could prevent cytoclasis and death induced by GF 109203X (Bisindolylmaleimide, an inhibitor of PKC). Furthermore, the results suggest that PKC, particularly PKCγ, plays an important role in the neuroprotective effects of EGCG [52]. More interestingly, a recent study demonstrated that EGCG could protect cells from stress damage by regulating extracellular signal-regulated kinase 1/2 (ERK1/2) and PKCα signaling pathways [53].

MAPKs are also serine/threonine kinases that participate in a variety of cellular activities including cell survival, death, proliferation, and differentiation [54]. MAPKs consist of three subfamilies including p38 MAPKs, the c-Jun NH2-terminal kinases (JNK) and ERK1/2 (p42/p44) [55]. ERK1/2 is involved in the regulation of cell proliferation and differentiation and p38 mediates inflammation and apoptosis, etc., while JNK responds to cellular stress such as radiation, osmotic pressure, oxidative stress, and temperature change [56,57]. Cyclic adenosine monophosphate (cAMP)-response element binding protein (CREB) plays an important regulatory role in neuronal generation, synaptic plasticity, learning, and memory and could mediate neuroprotective effects [58]. Schroeter et al. [59] showed that treatment with EC (100–300 nmol/L) in vitro stimulated AKT and ERK1/2 phosphorylation, which mediated CREB phosphorylation in neurons, thus modulating synaptic function and enhancing neuroprotection. Furthermore, a study showed that EGCG protected cells against oxyhaemoglobin-induced injury after subarachnoid haemorrhage via inhibition of the p38α pathway [60]. Moreover, 25 μmol/L EGCG may provide neuroprotection by inhibiting JNK activation and caspase 3 collapse in the thrombin-induced model of intracerebral hemorrhage [61]. Thus, the role of MAPKs signaling in neurodegenerative diseases is gradually emerging, indicating the manipulation of these signal transduction pathways as a potential strategy for therapeutic interventions [54,62].

PI3K/AKT has been proven to regulate various cell responses, such as cell proliferation, differentiation, and transformation [63,64]. Moreover, it participates in learning, memory, and synaptic plasticity [65,66,67]. In addition, oxidative stress occurs and the protein expression of acetylcholinesterase is increased by activating the PI3K/AKT signaling pathway of the central neurons with high levels of glucose [68]. In recent years, studies have found that the PI3K/AKT signaling pathway exerts enormous influence on the occurrence and development of neurodegenerative diseases [69,70]. For instance, EGCG could protect PC12, N18D3, or G93A mutant cells from oxidative stress through the PI3K/AKT signaling pathway [71,72,73]. Accordingly, a recent study found that EGCG in vitro (10 nmol/L) and in vivo (2.5 mg/kg) (the equivalent human dose is 0.27 mg/kg) could significantly improve neuronal survival and hippocampal neurogenesis by activating the PI3K/AKT signaling pathway [74]. In summary, there is an increasing amount of evidence revealing that TPs may play a neuroprotection role via activating the PI3K/AKT and PKC pathways and inhibiting the MAPKs pathway [24].

### 3.3. Metal Chelators Activity

Numerous studies have shown that the accumulation of metals such as iron, copper, and zinc is one of main pathological features of neurodegeneration diseases [75]. It is suspected that the metal (iron, copper, and zinc) environment in the cerebral cortex changes as the age increases and induces neurotoxicity, leading to neurodegenerative disease [76]. AD has two major pathological characters including Aβ and tau protein accumulation, however, PD is mainly characterized by aggregated α-synuclein within the pars compacta of the substantia nigra. In recent years, increasing evidence suggests that metal ions are involved in Aβ deposition and tau hyperphosphorylation, resulting in the formation of neurofibrillary tangles and amyloid plaques during AD development [77]. Many studies indicate that iron levels increase in the substantia nigra pars compacta (SNpc) of PD patients [78]. Therefore, metal chelators may be a novel clinical approach for AD and PD.

It has been demonstrated that EGCG has properties of metal chelation, as EGCG possesses 3’,4’-dihydroxyl and gallate groups in the B and C rings, respectively. Thus, ferric metal may be neutralized to an inactive form and may inhibit metal-induced oxidative stress that then protects the neuronal cells against damage [75,79]. It has been reported that EGCG has the ability to regulate amyloid precursor protein (APP) along with the presence of an iron responsive element and to reduce the toxic levels of Aβ [79,80]. Additionally, EGCG could protect mice from MPTP-induced neurotoxic damage by reducing iron and α-synuclein accumulation in SNpc [81]. Hung et al. [82] found that EGCG and other flavonoids have copper chelating properties by electrochemical measurements, and have potential as neuroprotective agents. In summary, EGCG may act as a metal chelator for AD and PD treatments.

## 4. Neuroprotective Effect and Mechanism of Theanine

Theanine is a unique amino acid of tea, accounting for 1–2% (dry weight) in tea fresh leaves. It has the positive health effects of improving learning and memory abilities, sedation, relaxation, anticancer properties, etc. [83,84]. Theanine can cross the blood brain barrier via the leucine-preferring transport system [85]. 

In recent years, numerous studies have shown that theanine has a neuroprotective effect. Nozawa et al. [86] found that 50% of neurons died in vitro when exposed to glutamate in a certain concentration. However, if pretreated with theanine, the probability of death was significantly decreased. Glutamate is the most abundant and widely available excitatory amino acid in the central nervous system and involves many important physiological functions in the brain. The increase in extracellular glutamate levels may induce a massive calcium ion influx and enhance formation of ROS, eventually leading to neuronal cell death [87]. In order to alleviate the toxicity of glutamate, the antagonists of glutamate receptors such as N-methyl-D-aspartate receptor (NMDAR) and α-amino-3-hydroxy-5-methyl-4-isoxazole-propionic acid receptor (AMPAR) have attracted more and more attention for neuroprotection in the last decade [88,89]. However, they have dose-limiting side effects [90]. Theanine, similar to glutamate in the chemical structure, can compete for the binding site of glutamate, such as NMDAR and AMPAR, thereby inhibiting the death of neurons. Consequently, theanine is a competitive antagonist of glutamate receptors [91,92]. Furthermore, Kakuda et al. [93] have found that theanine exerts inhibiting effects on the three glutamate receptors including NMDA, AMPA, and kainate receptor, suggesting it may provide neuroprotection, although the IC_50_ (50% inhibitory concentration) of theanine binding on NMDA receptor is about 10-fold lower than that of AMPA and kainate receptors. Zukhurova et al. [94] first demonstrated that theanine could protect rats from cerebral ischemia-reperfusion injury induced by glutamate receptor agonists, and the mechanism may be related to theanine as a glutamate receptor antagonist. These results show that theanine has neuroprotective effects by inhibiting the receptors of glutamate.

Theanine has an antagonistic effect to glutamate receptors, but the antagonistic effect is very weak [93]. Glutamine, derived from glutamate, is synthesized by glutamine synthetase. More recent evidence suggests that the glutamate-glutamine cycle formed between neurons and astrocytes is an important pathway for the regulation of glutamate concentration in the brain [95]. Theanine can inhibit the transport of glutamine and regulate the glutamate-glutamine cycle in the neurons [96] and, thus, provide neuroprotection. In addition, theanine could promote neurogenesis by increasing the expression of glutamine transporter slc38a1 [97,98]. The results further suggest that theanine may regulate the extracellular glutamine concentration in the neurons and by doing so may exert neuroprotective effects on neurodegenerative diseases.

Theanine is degraded to glutamate in normal cells, which increases the intracellular concentration of glutamine and glutathione, thus attenuating oxidative damage [99]. In addition, theanine pretreatment in PC12 cells reduces cadmium toxicity via antioxidant action [100]. Furthermore, pretreatment with theanine exerts neuroprotective effects against dopamine-induced [101] or 3-nitropropionic acid-induced [102] neurotoxicity, and the possible mechanisms are the increased levels of glutathione, which serves as an important free radical scavenger in the body, anti-inflammation, the preservation of striatal neurotransmitter homeostasis, and inhibition of the production of NO. In conclusion, theanine might be useful clinically as a neuroprotective agent in the future.

## 5. Neuroprotective Effects and Mechanisms of Caffeine

Caffeine is the dominant purine alkaloid in normal cultivated tea leaves and can stimulate the central nervous system [103]. Additionally, caffeine dose-dependently enhances cognitive and physical function by blocking adenosine receptors (ARs) [104]. ARs, including A_1_R, A_2A_R, A_2B_R, and A_3_R, are coupled to G-protein expressed in different cells. Furthermore, receptors of A_2A_R and A_2B_R are conjugated to stimulate Gs proteins, and receptors of A_1_ and A_3_ are conjugated to inhibit Gi proteins, respectively. Activation of these receptors leads to activation and inhibition of neural excitability, respectively [105,106]. Caffeine has a chemical structure very similar to adenosine, thus, it binds to adenosine receptors (particularly A_1_R and A_2A_R) as a competitive antagonist of adenosine. The inhibition of A_2A_R enhances the affinity of dopamine D_2_ ligands to D_2_ receptors and, furthermore, promotes the neurotransmission of dopamine. In addition to blocking adenosine receptors, caffeine has other effects at the cellular level, such as the inhibition of phosphodiesterase [107], modulation of calcium release from the intracellular calcium pool [108], and interference with GABA (γ-aminobutyric acid)-A receptors [109].

Numerous epidemiological and experimental studies have demonstrated that caffeine is negatively related to the incidence of neuro degenerative diseases [106]. Furthermore, the neuroprotection mechanism of caffeine has not been fully clarified, and the most likely mechanism depends on the ability of caffeine to antagonize A_2A_R. A series of studies have shown that A_2A_R antagonists have the ability to reduce Aβ-induced, aluminum chloride (AlCl_3_)-induced, or glutamate-induced toxicity and inhibit tau hyperphosphorylation which may be implicated in the progression of AD [110,111,112,113]. Furthermore, in a mouse AD model, activation of upregulated A_2A_R can cause the abolishment of long-term synaptic potentiation (LTP) in CA3 pyramidal cells, and A_2A_R antagonists revert the inhibition of LTP [114], which may provide theoretical support for the treatment of early AD patients. In addition, treatment with the antagonists of A_2A_R or A_2A_R deletion significantly improves memory in the THY-Tau22 mouse model [115]. As well, pretreatment with caffeine can protect SH-SY5Y cells against Aβ and AlCl_3_ induced damage due to the inhibition of mutual A_1_R and A_2A_R [110]. Furthermore, caffeine treatment is associated with the reduced tau pathology and improved memory in the THY-Tau22 mouse model [116].

Studies have shown that A_2A_R antagonists attenuated indirect-pathway spiny-projection neuron adaptations following dopamine depletion in PD models [117]. More recently, A_2A_R antagonists were shown to be involved in the control of α-synuclein aggregation and prevention of the associated neurotoxicity, which suggests that A_2A_R antagonists may act as potential drugs for PD treatment [118]. Furthermore, pretreatment with 25 mg/kg caffeine (the equivalent human dose is 2.75 mg/kg) significantly attenuates striatal degeneration and the loss of striatal dopamine in the MPTP model of PD, which is attributed to the A_2A_R [5,119]. Furthermore, both in vivo and in vitro studies have showed that caffeine and A_2A_R antagonists provide neuroprotection against neurovirulence induced by 6-OHDA [120,121]. Overall, caffeine as an A_2A_R antagonist may be a promising clinical agent in AD and PD.

The prominent pathology is axon degeneration in many neurodegenerative diseases, while the survival and growth of axons requires nicotinamide mononucleotide adenylyl transferase 2 (NMNAT2) [122], Furthermore, the levels of NMNAT2 are significantly decreased in AD [123]. Most recently, a study screening 1280 compounds with an NMNAT2-Meso Scale Discovery (MSD) platform showed that 24 compounds, including caffeine, had a significant impact on NMNAT2 levels, and NMNAT2 levels were upregulated by activating the cAMP signaling pathway or by exciting neurotransmission [124]. More interestingly, caffeine was identified as a positive modulator of NMNAT2 and restored the expression of NMNAT2 to normal levels in both in vitro and in vivo, thereby increasing neuronal viability [124].

## 6. Neuroprotective Effects and Mechanisms of Theaflavins

Theaflavins (TFs) are the main bioactive compounds of black tea, consisting of theaflavin (TF), theaflavin-3,3′-digallate (TFDiG), theaflavin-3′-gallate (TF3′G), and theaflavin-3-gallate (TF3G), which contribute to the color and quality of black tea. Furthermore, TFs have effective antioxidant properties resulting from free radical elimination and metal chelation [125,126]. Additionally, TFs possess many biological activities, such as anti-inflammation [127], anti-carcinogen [128], bacteriostat [129], and anti-viral [130].

In the last decade, many studies have shown that TFs have neuroprotective effects on neuronal degeneration diseases. TFs are at least equal in efficacy to EGCG at the inhibition of Aβ and α-synuclein induced toxicity due to their antioxidant properties [131]. Similarly, TFs protect PC12 cells from oxidative stress induced by H_2_O_2_ [132]. Furthermore, pretreatment with TFs at low concentrations prevented SH-SY5Y cell apoptosis induced by 6-OHDA, possibly by inhibiting the production of ROS and NO [133]. In addition, TFs have neuroprotective effects against MPTP-induced PD, which may be due to their abilities of antioxidation and antiapoptosis [134]. Based on the above results, we hypothesize that TFs have the possibility to be effective therapeutic agents for neurodegenerative diseases.

## 7. The Interaction between Tea Bioactive Components

At present, most studies are aimed at the effects of a single tea bioactive component or monomer on the nervous system alone. However, tea is composed of many interacting bioactive components, and a single tea bioactive component cannot fully reflect its normal physiological function. Therefore, it is essential to study the interaction between tea bioactive components. Dodd et al. [135] and Haskell et al. [136] have found that caffeine combined with theanine (amounting to 1–2 cups of tea) is beneficial for focusing attention and improving reaction speed and accuracy. Additionally, Park et al. [137] found that EGCG can inhibit fast/slow wave ratios induced by caffeine, which suggests that EGCG could reverse caffeine-induced anxiety. The synergistic mechanisms between catechin monomers and theanine, catechin monomers and caffeine, or theanine and caffeine remain to be elucidated.

## 8. Conclusions and Future Expectations

In vivo and in vitro studies have indicated that TPs, theanine, caffeine, and TFs show neuroprotective effects on neurodegenerative disease such as PD and AD. Most of the epidemiological investigations showed that drinking tea reduced the risk of cognitive impairment. Topics related to neuroprotection and the mechanisms of tea have been research hotspots in the field of tea and health research in recent years. Compared with western medicine, the neuroprotective effects of tea have the advantages of being multi-target, non-toxic, and having good synergistic effects. Because of the complex pathological mechanisms of neurodegenerative diseases, the multi-target therapy strategy has gradually become a trend, and the development of clinical drug synthesis is also multi-target role-oriented. Tea can play a neuroprotective role through multi-component interactions, but the research in this area is relatively less and not deep enough.

The development of a drug must pass through the cell stage and animal stage before entering the clinical trial stage. In general, because in vivo experiments are influenced by complex factors such as neuroendocrine and immunity, there is not necessarily a conversion relationship between in vitro and in vivo doses. Generally, the doses between humans and animals can be converted by body surface area calculation method, but there are also significant difficulties with respect to the large differences in drug resistance between humans and animals and between different animals. Moreover, Singh et al. [24] and Mandel et al. [138] have determined that the neuroprotective effects of EGCG are dose-dependent, and EGCG promotes cell survival at low doses, however, it promotes cell death at high doses. Similarly, caffeine consumption (3–5 mg/kg) could reduce the risk of AD and PD in both epidemiological and preclinical studies [106] and theanine at a high concentration (47.5 mg/day) could reduce the risk of dementia in the elderly [139]. However, it should be noted that there is still a long way to go to develop tea bioactive components as drugs for the treatment of neurodegenerative diseases because individual metabolic differences will lead to differential responses [23].

EGCG has several limitations, including poor stability, low solubility, and low bioavailability in the body, which substantially affects its efficacy [140]. There has been some research on improving the biological activity of EGCG by structural modification, including methylation modification, glycosylation modification, nano-carrier embedding, and other technologies, thereby achieving its targeted transport [141,142,143]. Caffeine is a commonly used nerve stimulant and food additive, and its pharmacological effects are extensive and complex. Furthermore, the intake of caffeine at different doses and at different times may produce different pharmacological effects, and sometimes even the opposite. Thus, further clinical studies are essential to investigate the therapeutic doses of caffeine in neurodegenerative diseases. In addition, the underlying mechanisms of caffeine need to be explored in depth [106]. Theanine has a neuroprotective effect, but the underlying mechanisms are not fully clarified. Therefore, further animal and clinical studies are necessary for understanding the mechanisms of theanine in neurodegenerative diseases, which can provide the theoretical basis for developing clinical medicine. At present, the neuroprotection of TFs is mostly based on the results from in vivo and in vitro studies, and many mechanisms of action need to be further studied. In addition, the application of TFs is mainly limited by instability and the difficulty of separation and purification. Therefore, investigating techniques of extraction and preparation would be beneficial to the development and utilization of TFs. To date, tea volatiles have been shown to contribute to positive mood, but the mechanisms need to be further studied. More importantly, tea volatile extraction methods must be developed, such as solid phase micro-extraction, simultaneous distillation and extraction, and steam distillation under reduced pressure, which would provide a material bases for the study of the effects of tea volatiles on the nervous system [12,144]. In summary, the in-depth study of these series of problems will further explain the neuroprotective effects and mechanisms of tea bioactive components.

## Figures and Tables

**Table 1:** Epidemiologic studies for tea consumption effects to neuro function.

Reference	Study Design	Location	Study Sample	Main Results
Tomata et al. [14]	Cohort study	Japan	5.7-year follow-up (13,645 participants aged 65 years or more)	Green tea consumption related to reduced dementia incidence, with interval (CI) (0.61–0.87)
Eskelinen et al. [21]	Population-based cohort study	North Karelia	1409 participants (71%) aged 65 to 79 years	Midlife tea consumption was not related to AD or dementia, while midlife coffee related to decreased AD or dementia risk
Kandinov et al. [15]	Population Cardiovascular Risk Factors, Aging, and Dementia (CAIDE) study	Tel Aviv Sourasky Medical Center, UK	278 patients who noticed first motor symptoms of PD（4 groups, aged <50, 51–60, 61–70, >70 years）	Tea consumption >3 cups/day delayed PD onset by 7.7 years
Ng et al. [16]	Cross-sectional study	Singapore	2398 adults aged ≥ 55 years	Regular drinking of tea, especially oolong tea or black tea, was related to better functional and physical performance
Feng et al. [17]	Cross-sectional study	Singapore	716 Chinese adults aged ≥ 55 years	Tea consumption was conductive to cognitive performance
Kuriyama et al. [18]	Cross-sectional study	Tsurugaya, Japan	1003 Japanese aged ≥70 years	Higher level green tea consumption was related to lower cognitive impairment
Ma et al. [19]	Meta-analysis	Asia, Europe, Australia, and USA	52, 503 participants aged ≥50 years	Tea drinking could decrease the attack of cognitive decline, cognitive impairment, and mild cognitive impairment in elderly
Qi and Li [20]	Meta-analysis	Asia, USA	Tea consumption（34,4895 participants）, caffeine consumption （49, 2 724 participants）aged 30–85 years	Tea consumption could decrease PD risk with a linear relation, and the consumption increment of caffeine (200 mg/day) and tea (2 cups/day) decreased PD risk by 17% and 26%, respectively
